# Oral Versus IV Treatment for Catheter-related Bloodstream Infections

**DOI:** 10.3201/eid1311.070729

**Published:** 2007-11

**Authors:** Burke A. Cunha

**Affiliations:** * Winthrop-University Hospital, Mineola, New York, USA; † State University of New York School of Medicine, Stony Brook, New York, USA

**Keywords:** Prevention, IV line infections, oral antibiotics, systemic infections, letter

**To the Editor:** I read with interest the article by Halton and Graves on the economics of catheter-related blood stream infections (*1*). The most important determinants of infection in a temporary central venous catheter (CVC) are location and duration (*2*). Also important are aseptic CVC insertion and maintenance. Reducing the economic effects of CVCs is important, but I believe clinicians should use oral antimicrobial agents more often in place of intravenous (IV) antimicrobial therapy.

The economic and clinical benefits of using oral versus intravenous antimicrobial therapy are considerable. Oral therapy has important advantages over intravenous therapy administered via CVCs. Clinical advantages of oral antimicrobial therapy include the elimination of phlebitis CVC line infections. At equivalent doses, acquisition costs of oral agents are less than intravenous counterparts. Healthcare institutions charge IV administration fees per antimicrobial intravenous dose. Administrative cost for intravenous antimicrobial agents is US $10/dose. Intravenous antimicrobial administration costs are eliminated when drugs are administered orally. In hospitalized patients, oral antimicrobial therapy results in a decreased hospital length of stay (LOS) with its attendant economic implications.

Oral therapy for serious systemic infections should be with high bioavailability drugs, i.e., >90%, which results in essentially the same serum/tissue levels as if when administered by IV. Because all parenteral antimicrobial agents do not have an oral formulation, clinicians should select an equivalent oral agent with the same spectrum as its parenteral counterpart to treat most serious systemic infections (*3*). Currently, oral antimicrobial agents are available to treat infections formerly only treatable with intravenous drugs, e.g., vancomycin-resistant enterococci, methicillin-resistant *Staphylococcal aureus*, and *Pseudomonas aeruginosa* (*4**,**5*).

Whenever possible, clinicians should opt for oral therapy instead of IV therapy. Oral antimicrobial therapy is not an initial option in critically ill patients requiring intravenous therapy and in those who are unable to absorb oral drugs. Fortunately, most patients are candidates for oral therapy or intravenous to oral switch therapy.

Substantial savings can be realized by using oral antimicrobial therapy initially or as soon as possible after initial IV therapy. The take-home message is, with the exception of critically ill patients and those unable to absorb oral drugs, clinicians should consider oral therapy before resorting too quickly to IV antimicrobial agents via CVC. Nosocomial (CVC) infections are important from a clinical and economic perspective. Clinicians should consider oral antimicrobial agents more frequently instead of having CVC lines placed for IV drug administration. Currently, oral agents are available to treat nearly all pathogens, even those formerly only treatable with intravenous antimicrobial drugs.
