# Supplementary material for: Oral Versus IV Treatment for Catheter-related Bloodstream Infections
Source: Emerg Infect Dis. 2007 Nov;13(11):1800–1. doi: 10.3201/eid1311.070729 (PMC2876959; doi:10.3201/eid1311.070729)
Supplement: Related texts

# Letter

**DOI:** 10.3201/eid1311.071046

**Published:** 2007-11

**Authors:** Kate Halton, Nicholas Graves

**Affiliations:** * Princess Alexandra Hospital, Brisbane, Queensland, Australia; † Queensland University of Technology, Brisbane, Queensland, Australia

## In Response

In his letter Cunha suggests that oral antimicrobial drug therapy is safer and less expensive than intravenous therapy via central venous catheters (CVCs) (*1*). CVCs are often used in critically ill patients to deliver antimicrobial drug therapy, but they expose patients to a risk of catheter-related bloodstream infection (CRBSI). Our current knowledge about the cost-effectiveness of allocating resources toward interventions that prevent CRBSI in patients requiring a CVC has already been reviewed (*2*). If antimicrobial drug therapy can be delivered orally for some patient groups, instead of through a CVC, then the costs and benefits of this alternate strategy should be evaluated.

Like any decision that involves the reallocation of resources toward a different clinical practice, this decision should not be based on instinct but subjected to a rigorous economic appraisal using a cost-effectiveness framework. The decision requires consideration of all relevant alternative modes of delivery, as identified with the help of clinical experts. Depending on the clinical context, options may include delivery via CVCs or peripheral lines, use of intravenous to oral switch therapy, or oral administration with a variety of dosing schedules.

To identify the most efficient mode of antimicrobial drug delivery, all relevant costs and benefits of each option should be specified and each mode of delivery compared in terms of a common outcome (e.g., the incremental cost per quality-adjusted life year). Financial costs or cost-savings are important, but not the sole consideration for a decision maker (*3*). Having identified the “best” option given our current understanding of the problem, we must then incorporate the residual uncertainty surrounding this choice into the evaluation, explore the level of confidence in the decision, and identify what future research is needed (*4*).

## References

[1] Cunha BA. Oral versus IV treatment for catheter-related bloodstream infections. Emerg Infect Dis. 2007;13:1800.1821757910.3201/eid1311.070729PMC2876959

[2] Halton K, Graves N. Economic evaluation and catheter-related bloodstream infections. Emerg Infect Dis. 2007;13:815–23.1755321810.3201/eid1306.070048PMC2792862

[3] Graves N, Halton K, Lairson D. Economics and preventing hospital-acquired infection—broadening the perspective. Infect Control Hosp Epidemiol. 2007;28:178–84. 10.1086/51078717265399

[4] Claxton K, Sculpher M, Drummond M. A rational framework for decision making by the National Institute for Clinical Excellence. Lancet. 2002;360:711–5. 10.1016/S0140-6736(02)09832-X12241891
